# Simpson’s Rubber—Price Reduced

**Published:** 1868-01

**Authors:** 


### SIMPSON'S RUBBER—PRICE REDUCED.

THE Dental gum manufactured under Simpson's patent, has recently been reduced from $8.00 per pound to $6.00. We feel well assured that the manufacturer of this gum is disposed to extend to the dental profession every facility and accommodation possible; and we have no hesitation in expressing the belief that it is equal if not superior to any dental rubber ever offered to the profession; and we recommend all to give it a trial, after which we think none other will be used. T.

